# Analysis of the surface structure and electrochemical properties of functionalized carbon nanodots as environmentally friendly corrosion inhibitors for maraging steel in hydrochloric acid

**DOI:** 10.1039/d5ra07512b

**Published:** 2025-11-18

**Authors:** Jyothi M. Alphonso, Ronald Aquin Nazareth, Geetha M. Pinto, Sudhakar Y. N.

**Affiliations:** a Department of Chemistry, Mahatma Gandhi Memorial College Udupi-574102 Karnataka India jyothialphonso305@gmail.com; b Department of Chemistry, St. Aloysius (Deemed to be University) Mangaluru-575003 Karnataka India ronald.nazareth@staloysius.edu.in geetha_pinto@staloysius.edu.in; c Department of Chemistry, Mangalore University Mangalagangotri Karnataka India; d Department of Chemistry, Manipal Institute of Technology, Manipal Academy of Higher Education Manipal 576104 India sudhakar.yn@manipal.edu

## Abstract

Maraging steel, although valued for its strength and durability, is highly prone to corrosion under acidic conditions. This work introduces functionalized carbon nanodots (FCNDs), synthesized from phenylalanine and citric acid, as novel, eco-friendly corrosion inhibitors for maraging steel in 1.0 M hydrochloric acid. Electrochemical techniques, including Tafel polarization and EIS, were used to evaluate the inhibition performance at various FCND concentrations and temperatures. The results clearly show an increase in the inhibition efficiency with increasing FCND dosage, although elevated temperatures and acid strengths reduce the effectiveness. Adsorption studies confirmed a Langmuir-type behavior governed mainly by physisorption. This study not only introduces carbon nanodots as a promising class of green corrosion inhibitors but also establishes their potential applicability in protecting high-strength maraging steel components exposed to acidic industrial environments.

## Abstract 2

Functionalized carbon nanodots synthesized from phenylalanine and citric acid as corrosion inhibitors for maraging steel.

## Graphical abstract

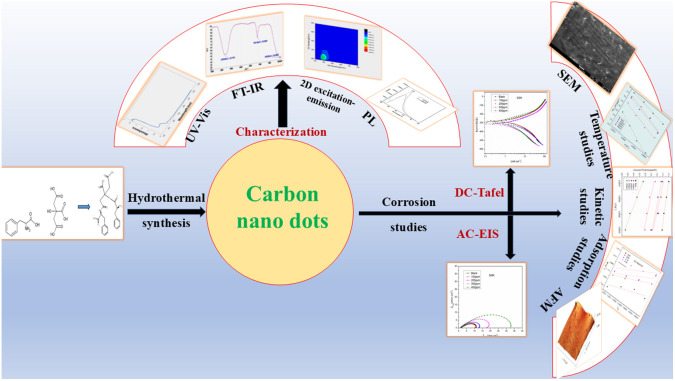

## 1. Introduction

Metals and their alloys serve as indispensable components in modern engineering because of their robust mechanical characteristics, ease of fabrication, and broad commercial availability. These materials find application in sectors such as civil infrastructure, automotive production, industrial equipment, and transportation systems. However, in hostile environments, metals are susceptible to degradation through corrosion—a natural but destructive phenomenon that gradually diminishes their structural integrity.^1^

The widespread occurrence of metal corrosion has significant implications, not only in terms of economic loss but also concerning health and safety within the industrial and public sectors. Although this process cannot be completely avoided, its progression can be effectively managed through suitable mitigation strategies.^2^ Corrosion typically results from electrochemical reactions between a metallic surface and its surrounding medium. For example, the corrosion of iron leads to the formation of reddish-brown hydrated ferric oxide, a product of electron and mass transfer at the metal–electrolyte interface.^3^ There are multiple techniques to avoid corrosion, such as cathodic and anodic protection, alloying, surface coating, and the use of chemical inhibitors. Among these methods, the use of corrosion inhibitors is widely adopted because of their cost-effectiveness and relative ease of application.^4,5^ In recent years, graphene oxide-based materials have garnered significant attention as potential corrosion-resistant coatings because of their large surface area, thermal stability, stable mechanical properties, and impermeability to water, oxygen molecules, and ions.^6^ Many inhibitors work by adhering to the metal surface, forming a barrier that limits direct interaction with corrosive agents. Organic inhibitors containing elements such as nitrogen, sulfur, or oxygen are particularly efficient owing to their ability to coordinate with metal ions and form stable protective films.^7,8^

Corrosion can occur in various forms, depending on the environmental conditions and the nature of the exposed material. Typical classifications include erosion–corrosion, selective leaching, stress corrosion cracking, uniform deterioration, localized pitting, grain boundary corrosion, corrosion in crevices, and galvanic effects.^9–11^ One effective approach to reduce corrosion is the use of metallic alloys. These materials often experience selective surface enrichment of more noble elements, which enhances their corrosion resistance. A particularly notable alloy in this context is maraging steel, which is characterized by excellent strength, toughness, and dimensional stability due to its unique microstructure.^12^ Maraging steels are distinct from conventional steels in that their hardness does not originate from the carbon content but rather from the precipitation of intermetallic phases during the thermal aging process. This aging, which occurs within a martensitic matrix, significantly enhances the mechanical properties while maintaining good formability and weldability.^13,14^

In recent years, growing environmental concerns and regulatory restrictions on toxic organic inhibitors have led to increased interest in environmentally benign alternatives. Among these materials, carbon dots (CDs)—a category of zero-dimensional nanostructures—have garnered attention for their promising characteristics. With average sizes below 20 nm (although sometimes reaching up to 60 nm), CDs offer features such as strong photoluminescence, high water solubility, a large surface area, tunable functional groups, and excellent biocompatibility.^15–17^ These carbon-based nanomaterials were first discovered during the purification of carbon nanotubes by Xu and colleagues,^18^ and since then, their potential applications have expanded into fields such as biomedicine, sensing, and electrochemistry. The corrosion inhibition properties of CDs have recently been explored, with encouraging results. For example, Shuyun Cao *et al.* demonstrated that nitrogen-doped CDs (N-CDs) provided 97.8% inhibition efficiency for carbon steel in sulfuric acid at 298 K when only 30 mg L^−1^ of the inhibitor was used.^19^ Similarly, Mingjun Cui and coworkers investigated the performance of CDs on Q235 steel in hydrochloric acid and reported a significant reduction in corrosion rates.^20^ In another study, Dongping Yang *et al.* synthesized CDs using imidazole ionic liquids and citric acid and reported that the inhibitors were more effective in 1 M HCl than in 3.5 wt% NaCl solutions, reflecting their performance across various corrosive environments.^21^ Further studies revealed the role of heteroatom doping in enhancing inhibition. Vandana Saraswat *et al.* reported an inhibition efficiency of 98.64% on mild steel using nitrogen and sulphur codoped CDs at 200 mg L^−1^ and 303 K.^22^ Hongyu Cen and team examined the protection of aluminum alloys in hydrochloric acid and achieved 85.9% efficiency with just 5 mg L^−1^ N^−1^, S-CD.^23^ Additionally, Subash Padhan *et al.* introduced copper into nitrogen-doped CDs and demonstrated high inhibition even at low concentrations, emphasizing the beneficial effects of metal ion doping.^24^ Despite these promising advances, research involving CDs as corrosion inhibitors for maraging steel remains scarce. Given the strategic importance of maraging steels in aerospace and high-performance structural applications, there is a compelling need to explore sustainable inhibition strategies tailored for such materials. The present study addresses this gap by investigating the use of functionalized carbon nanodots as corrosion inhibitors for maraging steel, with a focus on their adsorption behavior and protective performance in corrosive environments.

## 2. Experimental

### 2.1 Materials and chemicals

M250-grade maraging steel was obtained from a local shop, and phenylalanine and citric acid were procured from Merck.

### 2.2 Method

The study utilized an aged M250-grade maraging steel sample shaped into a cylindrical rod. The elemental composition (wt%) of the 18Ni 250-grade maraging steel is shown in Table 1.

**Table 1:** Composition of the maraging steel

Element	% composition
Ni	18.19
Co	7.84
Mo	4.82
Ti	0.52
C	0.015
Fe	Balance

**Scheme 1:**
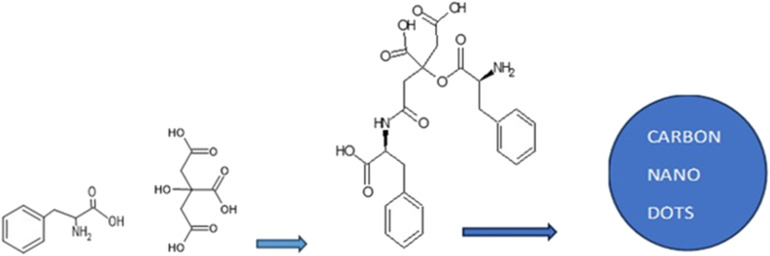
Illustration of the initial reaction steps during the synthesis of carbon nanodots from citric acid and phenylalanine.^25^

The functionalized carbon nanodot (FCND) inhibitor was synthesized on the basis of previously reported procedures.^23^ Fifteen milliliters of water was used to dissolve phenylalanine and citric acid, which were then hydrothermally processed for eight hours at 200 °C in an autoclave lined with Teflon. After the suspension naturally cooled to room temperature, it was filtered. The carbon nanodots were designated according to the mole fraction of phenylalanine used in the mixture. For this work, P75, containing 75 mol% phenylalanine and 25 mol% citric acid, was selected (Scheme 1).

### 2.3 Electrochemical study

The corrosive medium was prepared by dissolving hydrochloric acid to obtain a 1.0 M concentration, which was used to prepare inhibitor (FCND) solutions of varying concentrations. Electrochemical experiments were carried out with a saturated calomel electrode (SCE) as the reference, a platinum electrode as the counter, and a maraging steel sample as the working electrode assembled in a Pyrex glass cell. The electrochemical measurements were conducted using an ACM Instrument electrochemical workstation (Gill AC, Serial No. 1726, UK).

The maraging steel sample was sectioned from a larger plate and mounted in epoxy resin, leaving a defined area exposed for electrochemical testing. The exposed surface was initially abraded with Emery papers ranging from 150 to 2000 grit and then subjected to fine polishing on a polishing wheel using an alumina slurry to achieve a mirror-like finish. The polished sample was subsequently cleaned with acetone, rinsed thoroughly with double-distilled water, and finally conditioned by washing with the test electrolyte solution. The effective surface area of the maraging steel electrode exposed during the EIS and PDP measurements was precisely maintained at 0.92 cm^2^. Measurements were taken by applying a sinusoidal voltage of 10 mV at the system's open circuit potential (OCP), covering a frequency range from 100 kHz to 0.01 Hz. The resulting impedance profiles were examined through Nyquist plots, and the charge transfer resistance (*R*_ct_) was determined from the size of the notable semicircular section of the plots. To ensure the reliability of the data, each experiment was repeated at least three times, with the average values of the key parameters being used for interpretation.

### 2.4 Characterization techniques

Using scanning electron microscopy in conjunction with energy-dispersive X-ray spectroscopy (SEM-EDS), the surface morphology of the maraging steel samples was investigated both before and after they were exposed to an acidic environment, both with and without the inhibitor. Instruments from 7610FPLUS, Jeol, Japan, and GEMINI 300, Carl Zeiss, Germany, were used for the analyses. UV-visible absorbance readings were recorded *via* a SYSTRONICS PC-based double beam Spectrophotometer 2202, with Serial No. 445. Infrared spectroscopy measurements *via* Fourier transform were conducted with a PerkinElmer Spectrum IR 10.7.2. The fluorescence properties were evaluated *via* two instruments: a Horiba spectrofluorometer (Model: FLUOROMAX_PLUS_C_0844G-3324-FMPLUS R928P) and an FLS1000 system from Edinburgh Instruments, UK. Topographical imaging through atomic force microscopy (AFM) was carried out in tapping mode using the Flex-Axiom system from NanoSurf, Switzerland.

## 3. Results and discussion

### 3.1 Characterization

**Fig. 1:**
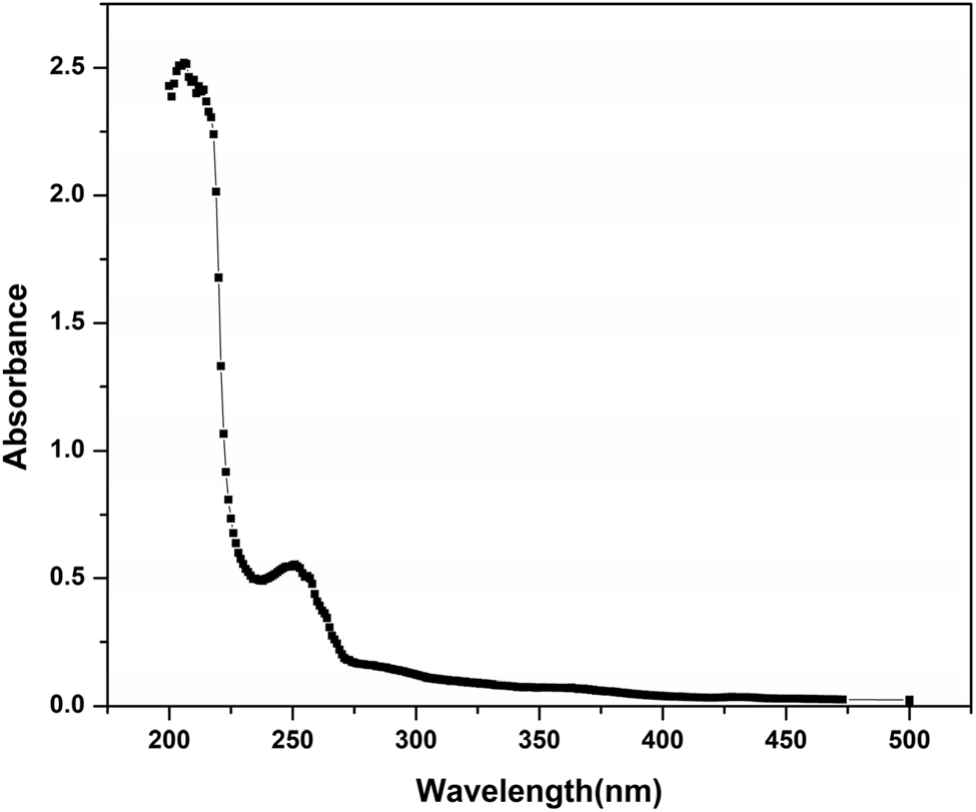
Absorbance spectra of the P_75_ CDs.

The UV-visible absorption profile of the prepared FCNDs, measured between 200 and 500 nm, revealed a strong absorption band at 210 nm and a secondary feature at approximately 250 nm. A gradual absorption extension into the visible range was also noted, likely arising from π–π* electronic transitions of C=C moieties and n–π transitions linked to C=O functionalities,^26^ as depicted in Fig. 1.

**Fig. 2:**
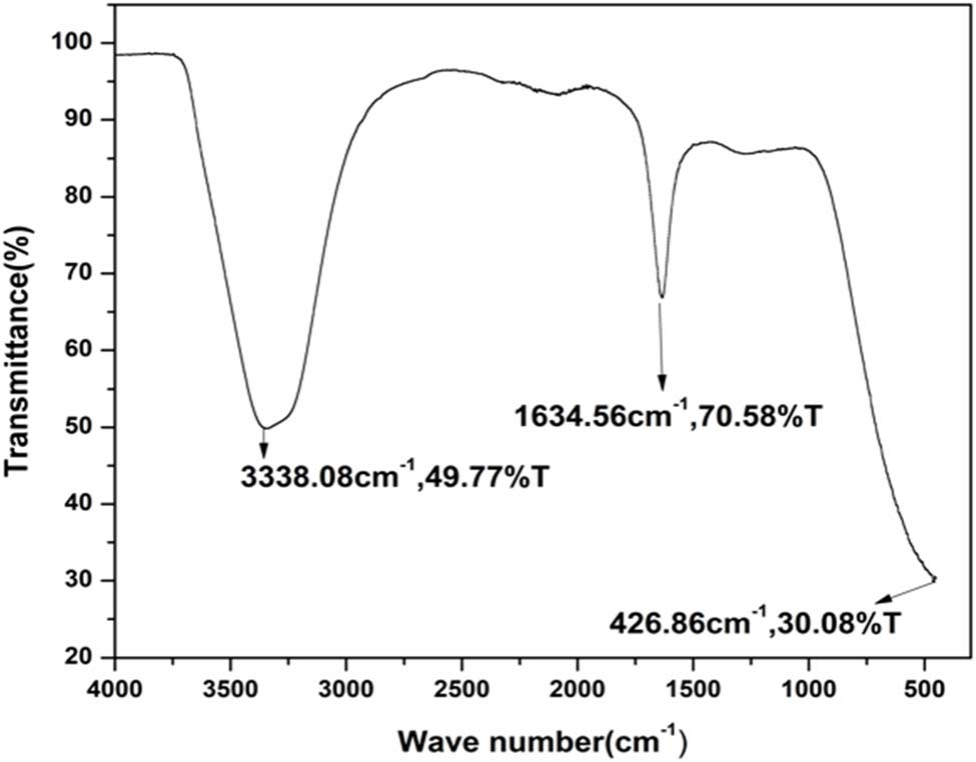
FTIR spectrum of the P_75_ CDs.

The FTIR spectra provide strong evidence for the attachment of amino acid-derived functional groups to the surface of the carbon dots. The distinct and intense absorption band observed within the range of 1600–1800 cm^−1^ is attributed to the vibrational stretching modes of carbonyl-related groups, including carboxylate, carbonyl, and amide functionalities. Furthermore, a broad absorption feature appearing between 3200 and 3500 cm^−1^ indicates the presence of hydroxyl (–OH) and amine (–NH_2_) groups, confirming the surface functionalization of the synthesized C-dots, as presented in Fig. 2.^27^

**Fig. 3:**
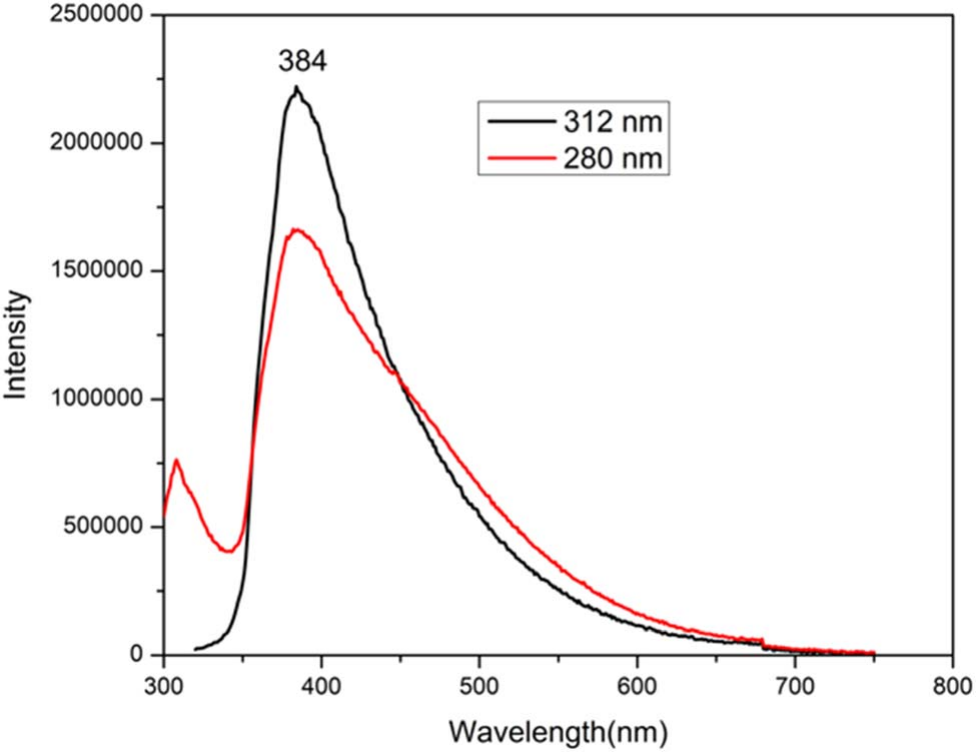
PL spectra of FCND at 384 nm and 280 nm excitation.

A systematic photoluminescence (PL) study was performed to better understand the optical behavior of the synthesized FCNDs using a range of excitation wavelengths (Fig. 3). As the excitation wavelength decreased from 384 nm to 280 nm, the PL intensity decreased significantly; however, the emission wavelength remained constant. This wavelength-independent PL response is in stark contrast to many carbon-based nanomaterials reported earlier,^28–30^ which typically show a redshift in emission with increasing excitation wavelength. The consistent emission feature observed here is likely due to the highly uniform size distribution and minimal surface defects present in the FCNDs.^31^

#### 3.1.1 Excitation–emission matrix (EEM) analysis of carbon dots

**Fig. 4:**
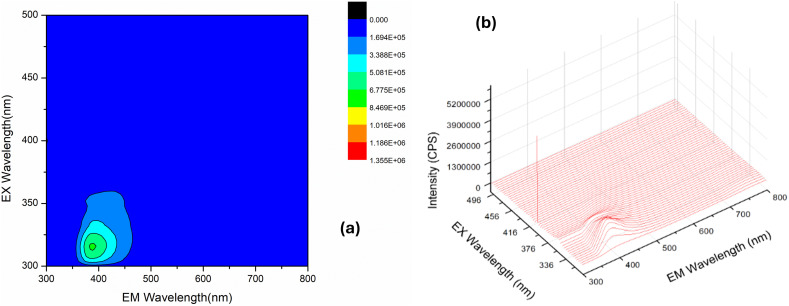
(a) 2D excitation-emission map of FCND, (b) 2D contour map of FCND.

The contour plot displays the excitation–emission matrix for a functionalized carbon nanodot (FCND) sample dispersed in water, highlighting its photoluminescent properties. A detailed excitation–emission matrix (EEM) analysis was conducted to further elucidate the photoluminescent properties of the FCNDs. As shown in Fig. 4a, a prominent luminescence region appears around an excitation wavelength of approximately 315 nm and a corresponding emission near 395 nm, marked by the most concentrated contour lines.^32^

#### 3.1.2 Three-dimensional fluorescence surface plot

The 3D fluorescence graph shows how the emission intensity (measured in counts per second, CPS) changes with varying excitation (EX) and emission (EM) wavelengths for the functionalized carbon nanodots (FCNDs) (Fig. 4b). A prominent emission range appears between 325 nm and 425 nm on the emission axis, highlighting the specific wavelengths where the CDs demonstrate strong photoluminescent behavior.

#### 3.1.3 SEM/EDS analysis

**Fig. 5:**
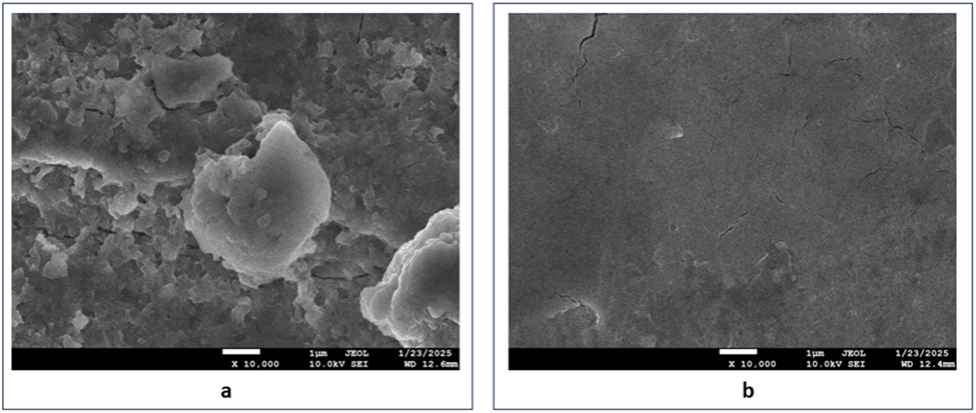
SEM images of aged maraging steel welds after being immersed in 1.0 M hydrochloric acid: (a) without the presence of FCND and (b) with the presence of FCND.

Fig. 5a presents an SEM image of the weld-aged maraging steel surface after immersion in a 1.0 M hydrochloric acid solution in the absence of functionalized carbon nanodots (FCNDs). The image reveals extensive surface damage characterized by visible pits, deposits, and irregular textures, indicating pronounced corrosion activity. This deterioration suggests active interactions between the metal surface and the acidic medium, leading to localized material degradation. On the other hand, Fig. 5b shows the surface morphology of the steel sample treated under identical conditions in the presence of FCNDs. A marked improvement in surface integrity is evident, with the sample exhibiting a smoother texture and a substantial reduction in visible corrosion features. The suppression of pit formation and preservation of surface uniformity indicate that the FCNDs effectively adhered to the metal surface, acting as a barrier to corrosive species and minimizing the extent of acid-induced attack. These morphological differences affirm the protective capability of fluorescent carbon nanodots, suggesting their potential role as efficient corrosion-inhibiting agents for maraging steel in aggressive acidic environments.^33,34^

**Fig. 6:**
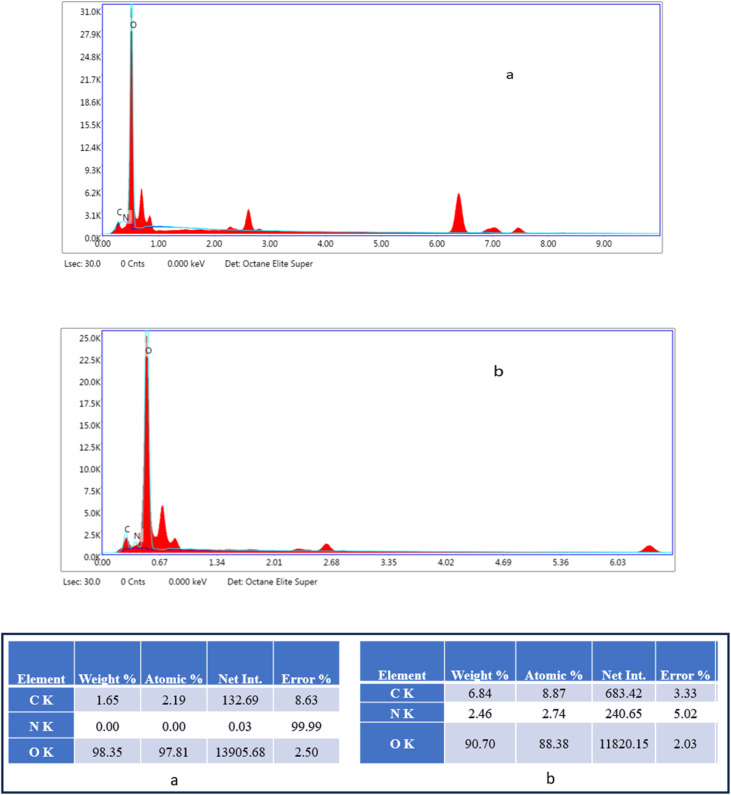
EDS spectra of weld-aged maraging steel following exposure to 1.0 M hydrochloric acid: (a) without FCNDs and (b) with FCNDs incorporated.

In Fig. 6a, the carbon signal is minimal (1.65 wt%), which aligns with the trace amount of carbon naturally present in maraging steel. No nitrogen signal was detected, whereas oxygen dominated the spectrum (98.35 wt%), implying substantial oxidation and corrosion on the metal surface due to the aggressive acidic environment. In contrast, Fig. 6b shows a notable increase in the carbon content (6.84 wt%) and a detectable amount of nitrogen (2.46 wt%), with oxygen reduced slightly to 90.70 wt%. The higher carbon concentration confirmed that the phenylalanine-derived carbon dots were successfully deposited onto the steel surface. The appearance of nitrogen in this set suggests that nitrogen-containing functional groups from the amino acid precursor remained attached to the surface, likely contributing to the formation of a protective barrier. These elemental differences highlight the role of carbon dots in mitigating acid-induced corrosion by establishing a chemically interactive coating on the steel substrate.

### 3.2 Corrosion inhibition study

Electrochemical investigations, potentiodynamic polarization (PDP) and EIS analyses, were carried out at various temperatures: 303 K, 308 K, 313 K and 318 K.

### 3.3 DC electrochemical techniques – potentiodynamic polarization (PDP) studies using Tafel extrapolation

**Fig. 7:**
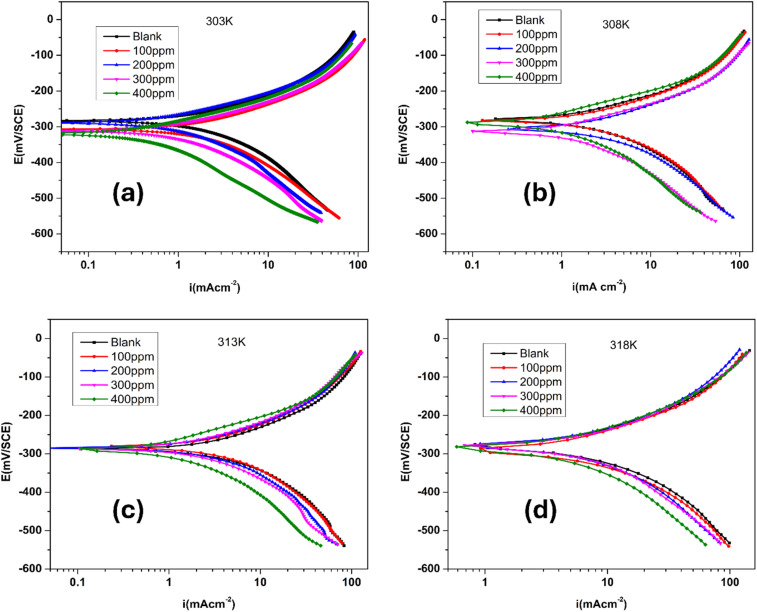
Tafel plots of maraging steel immersed in a 1 M HCl solution containing various concentrations of functionalized carbon nanodots (FCNDs) at four different temperatures: (a) 303 K, (b) 308 K, (c) 313 K, and (d) 318 K.

Polished alloy samples were immersed in corrosive media with varying concentrations and subjected to different temperature conditions ranging from 303 K to 318 K. After achieving a stable open circuit potential (OCP), the samples underwent polarization by adjusting the potential from −250 mV to +250 mV relative to the OCP at a scan rate of 1 mV s^−1^. The resulting current–potential curves were examined *via* the Tafel extrapolation technique to obtain important polarization parameters. Potentiodynamic polarization curves for the corrosion of maraging steel in 1.0 M HCl media with various concentrations of the FCND inhibitor at 303 K, 308 K, 313 K and 318 K are shown in Fig. 7.

A slight variation in the corrosion potential (*E*_corr_) suggests that the functionalized carbon nanodots (FCNDs) exhibit the characteristics of a mixed-type corrosion inhibitor. This implies that FCNDs influence both anodic metal dissolution and cathodic hydrogen evolution processes during corrosion. With increasing concentrations of FCNDs, a notable decline in corrosion current density (*i*_corr_) is observed, indicating enhanced corrosion resistance. The electrochemical parameters, including *i*_corr_, *E*_corr_, and Tafel slopes (*β*_a_ and *β*_c_), are summarized in Table 2.

**Table 2:** Results of potentodynamic polarization studies on maraging steel in 1.0 M HCl containing different concentrations of functionalised carbon nanodots

Temperature (K)	Inhibitor concentration (ppm)	*E*_corr_ (mV/SCE)	*β*_a_ (mV)	−*β*_c_ (mV)	*I*_corr_ (mA cm^−2^) ± SD	*γ*_corr_ (mm per year)	*η* (%) ± SD
303	Blank	−316.3	113.0	164.9	6.8147 ± 0.0006	92.071	
	100	−313.4	97.4	160.4	3.8056 ± 0.0051	51.432	44.12 ± 0.02
	200	−305.32	94.4	162.3	3.6909 ± 0.0009	49.875	45.84 ± 0.04
	300	−290.38	87.6	145.3	3.2688 ± 0.0020	43.211	52.04 ± 0.05
	400	−284.44	88.9	141.8	1.8413 ± 0.0107	24.236	73.07 ± 0.11
308	Blank	−313.76	126.9	171.9	5.5086 ± 0.0072	74.411	
	100	−303.41	130.7	175.7	4.8580 ± 0.0044	65.604	11.82 ± 0.02
	200	−289.51	120.0	154.5	4.5368 ± 0.0021	64.237	17.57 ± 0.06
	300	−283.71	97.6	158.7	3.0387 ± 0.0263	41.314	44.76 ± 0.05
	400	−279.99	98.4	179.7	1.5523 ± 0.0060	21.227	71.67 ± 0.06
313	Blank	−289.11	157.1	193.4	5.9348 ± 0.0053	80.146	
	100	−288.85	141.7	192.3	5.3796 ± 0.0133	72.789	9.16 ± 0.05
	200	−285.54	141.9	171.5	4.7804 ± 0.0134	64.721	19.25 ± 0.05
	300	−284.64	106.1	166.4	4.4148 ± 0.0089	59.542	25.67 ± 0.06
	400	−282.38	130.2	209.2	2.3610 ± 0.0221	31.989	60.44 ± 0.04
318	Blank	−290.25	175.8	205.4	6.6067 ± 0.0064	89.217	
	100	−286.45	152.3	160.2	5.8225 ± 0.0123	78.023	12.07 ± 0.10
	200	−282.96	164.6	207.5	5.4950 ± 0.0079	74.298	16.73 ± 0.06
	300	−281.4	140.5	187.4	3.7153 ± 0.0140	50.045	43.86 ± 0.14
	400	−279.1	138.2	201.9	3.0226 ± 0.0035	40.246	54.16 ± 0.05

The variability in *β*_c_ values is minimal. This suggests that the inhibitor does not affect the hydrogen evolution pathway. Inhibition is achieved solely through adsorption, which blocks the surface.^35^ Changes in the inhibitor concentration have little effect on the *β*_a_ and *β*_c_ Tafel slopes, which remain largely unchanged, indicating that the fundamental anodic and cathodic reaction pathways are not significantly altered by the presence of FCNDs.^36,37^ Instead, the observed inhibition is attributed to the surface coverage or barrier effect offered by the adsorbed FCNDs. This protective layer limits the access of corrosive species to the metal surface, thereby suppressing both anodic and cathodic reactions. On the basis of these observations, FCNDs can be classified as mixed-type inhibitors,^38^ functioning primarily through adsorption and the formation of a passive protective film on the alloy surface.

**Fig. 8:**
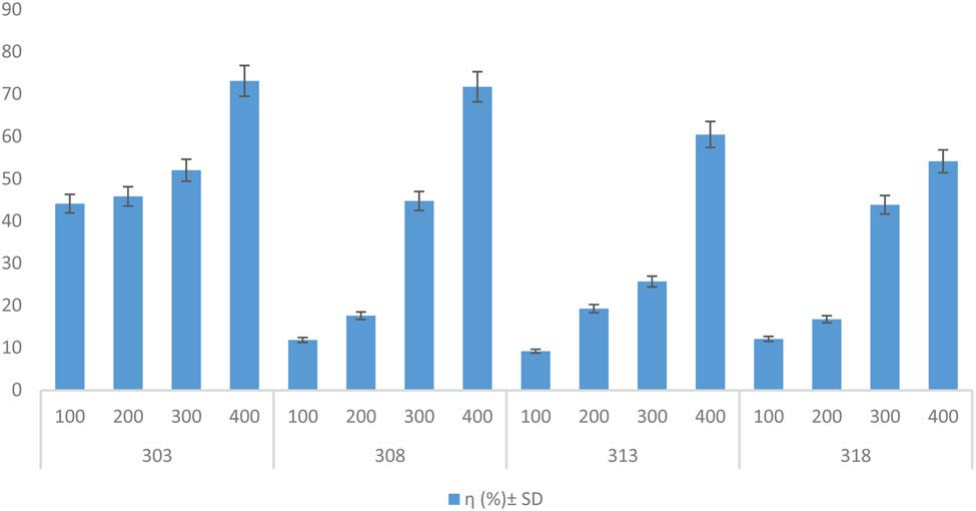
Error bars of the inhibition efficiency *versus* the inhibitor concentration, indicating minimal experimental error.

Adding error bars to the primary graph increased the certainty of the data trend. By displaying error bars for each data point, the plot provides visual evidence of how consistent the experimental measurements are. The very small spread of the bars shows that the variation in the data is minimal, indicating that the trend observed between the inhibitor concentration and inhibition efficiency is dependable and not due to random measurement fluctuations (Fig. 8).

The slopes of the anodic and cathodic branches of the Tafel curves change as the concentration of FCNDs increases. These findings suggest that the FCNDs affect both anodic and cathodic processes, reducing the degree of oxidation of the metal as well as the degree of hydrogen evolution on the steel surface.

The corrosion rate (mm per year), CR, was calculated *via*eqn (1):1
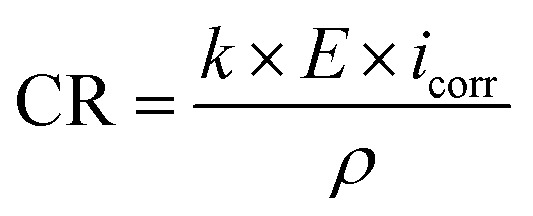


The constant *k* is 3.270 × 10^−3^ mm g (mA cm year)^−1^, the alloy's equivalent weight is denoted by *E*, its corrosion current density is quantified in units of mA cm^−2^ and symbolized by *i*_corr_, and its density is expressed in units of kg m^−3^ marked by *ρ*. To determine the corrosion inhibition efficiency *η* (%), eqn (2) was used.2
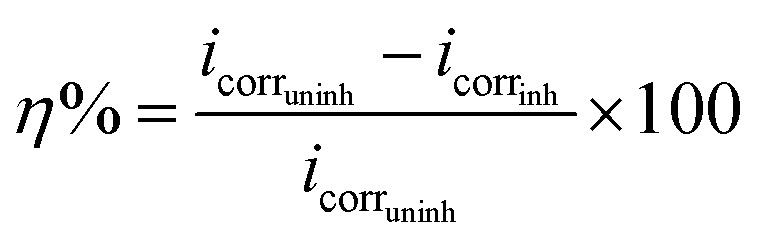


### 3.4 AC electrochemical techniques–electrochemical impedance spectroscopy (EIS) analysis

The Nyquist plots highlighted the effects of varying doses of FCNDs synthesized from citric acid and phenylalanine on the corrosion behavior of maraging steel in 1.0 M HCl at 303 K. The values of the impedance obtained with and without the inhibitor are denoted as *R*_ctinh_ and *R*_ctuninh_, respectively. An increase in *R*_ct_ under inhibited conditions suggests a significant retardation of the charge transfer mechanism at the metal–solution interface, which is indicative of protective film formation by the FCNDs. This increase is typically linked to a greater interfacial barrier, possibly due to a denser inhibitor layer and/or a decrease in the local dielectric constant, both of which result in a reduced double-layer capacitance (*C*_dl_).^39^

The effectiveness of inhibition (*η*%) was determined *via* the relationship outlined below: 3
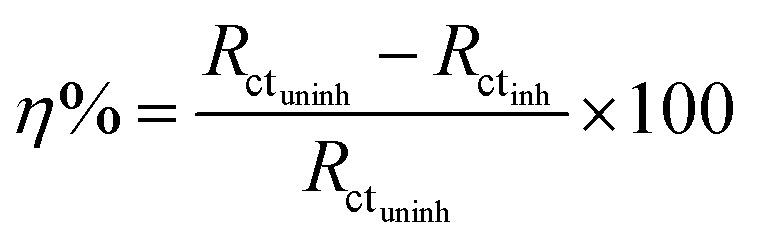


The electrical double layer capacitance, *C*_dl_, at the interface was determined *via* the following equation:4
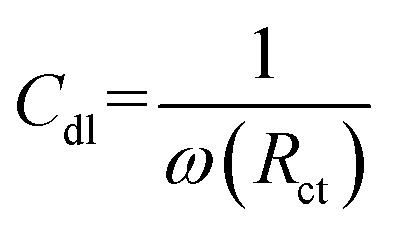
*ω* refers to the angular frequency and is calculated *via* the expression *ω* = 2π*f*, where *f* represents the frequency at which the imaginary part of the impedance reaches its maximum value. A progressive decrease in *C*_dl_ with increasing inhibitor concentration reflects a more compact and less conductive double layer, which restricts ion transport and thus enhances corrosion protection.^40^

### 3.5 Interpretation of the Nyquist plot

**Fig. 9:**
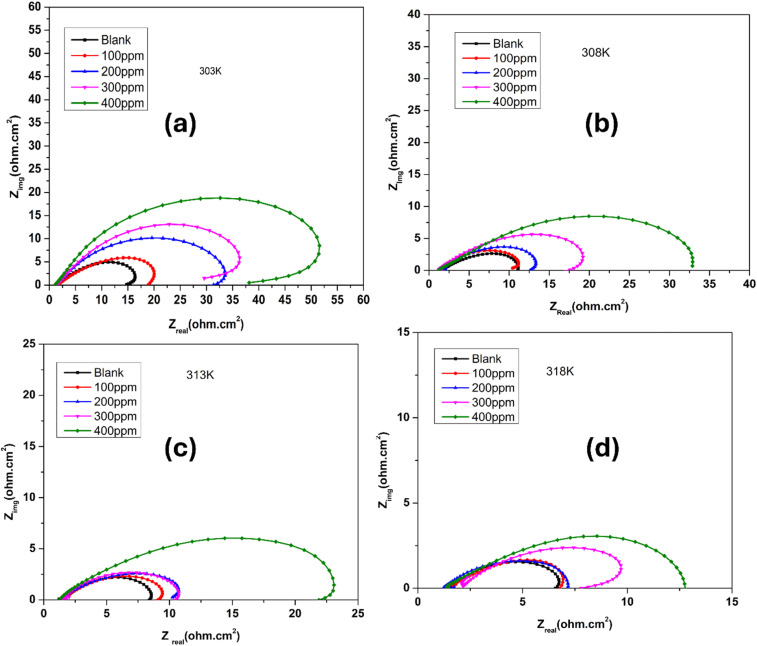
Nyquist plots of steel immersed in a 1 M HCl solution containing various concentrations of functionalized carbon nanodots (FCNDs) at four different temperatures: (a) 303 K, (b) 308 K, (c) 313 K and (d) 318 K.

Electrochemical impedance spectroscopy (EIS) was used to assess the corrosion characteristics of maraging steel in a hydrochloric acid environment, both without and with functionalized carbon nanodots (FCNDs), at four distinct temperatures: 303 K, 308 K, 313 K, and 318 K. The related Nyquist plots are shown in Fig. 9. In the impedance diagram, the *X*-direction corresponds to *Z*′, the real impedance, whereas the *Y*-direction reflects *Z*″, the imaginary counterpart. The impedance spectra display semicircular arcs, which are indicative of charge transfer processes at the metal–electrolyte interface. This semicircular nature is commonly associated with electrochemical systems undergoing corrosion. The appearance of capacitive loops at high frequencies and inductive loops at lower frequencies is typical for these systems, whether inhibited or uninhibited. A clear trend emerges from the plots: as the FCND concentration increases, the diameter of the semicircle becomes larger. This expansion reflects a higher charge transfer resistance, implying improved resistance to corrosion. The protective benefit is linked to the adsorption of FCNDs onto the surface of the steel, creating an insulating layer that restricts the interaction between the metal and the corrosive environment.

The blank sample (represented by the black curve), which contains no inhibitor, has the smallest semicircle and the lowest impedance values, indicating rapid corrosion. As the FCND concentration increases from 100 ppm (red curve) to 400 ppm (green curve), the size of the semicircles significantly increases. This observation suggests that higher concentrations of FCNDs enhance the inhibition efficiency by strengthening the surface barrier. Furthermore, the slightly flattened shape of the semicircles suggests surface irregularities and nonuniform adsorption, leading to frequency dispersion—an effect commonly observed on solid metal surfaces with heterogeneous morphology.^41^ The elevated *R*_ct_ values in the presence of FCNDs support the conclusion that these nanodots act as effective corrosion inhibitors by reducing the rate of electrochemical reactions on the steel surface.^42–44^

Table 3 clearly shows that as the inhibitor concentration increases, the CPE values tend to decrease, whereas the *R*_ct_ values increase. The decrease in the CPE can be linked to an increase in the electrical double layer thickness or a change in the local dielectric constant, indicating that the FCND operates through adsorption at the metal/solution interface.^45^ As the inhibitor concentration increased, more FCND particles adsorbed on the surface, resulting in lower CPE values and a thicker film.

**Table 3:** Electrochemical impedance spectroscopy (EIS) results for maraging steel exposed to 1.0 M HCl with varying amounts of functionalized carbon nanodots

Temperature (K)	Inhibitor concentration (ppm)	*R*_ct_ (Ω cm^2^) ±SD	*C*_dl_ (mF cm^−2^) ± SD	*R*_sol_ (Ω cm^2^)	CPE, Ω^−1^ cm^−2^	*η* (%)
303	Blank	15.28 ± 0.03	8.94 ± 0.006	1.207	0.99	
	100	27.11 ± 0.02	4.975 ± 0.005	1.64	0.93	43.65
	200	28.42 ± 0.03	3.037 ± 0.006	1.169	0.91	46.24
	300	32.13 ± 0.05	2.925 ± 0.005	1.349	0.83	52.45
	400	56.70 ± 0.10	1.387 ± 0.006	1.2	0.76	73.06
308	Blank	7.235 ± 0.003	16.847 ± 0.040	1.649	1.13	
	100	8.178 ± 0.007	6.526 ± 0.005	1.238	1,12	11.56
	200	8.800 ± 0.020	5.719 ± 0.026	1.563	1.11	17.93
	300	13.090 ± 0.017	5.638 ± 0.005	1.357	0.85	44.62
	400	25.200 ± 0.100	2.177 ± 0.006	1.269	0.84	71.27
313	Blank	5.027 ± 0.022	4.944 ± 0.006	1.249	1.14	
	100	5.576 ± 0.004	4.929 ± 0.010	1.676	1.13	9.61
	200	6.257 ± 0.006	5.846 ± 0.005	1.646	1.11	19.48
	300	6.745 ± 0.005	3.155 ± 0.005	1.935	1.05	25.24
	400	12.700 ± 0.100	3.238 ± 0.027	1.294	1.08	60.61
318	Blank	3.787 ± 0.006	11.687 ± 0.104	1.473	1.20	
	100	4.337 ± 0.006	8.942 ± 0.034	1.732	1.15	12.67
	200	4.538 ± 0.052	8.044 ± 0.036	1.588	1.12	16.04
	300	6.724 ± 0.005	6.945 ± 0.045	2.117	1.08	43.61
	400	8.395 ± 0.005	5.831 ± 0.052	1.447	1.00	54.84

**Fig. 10:**
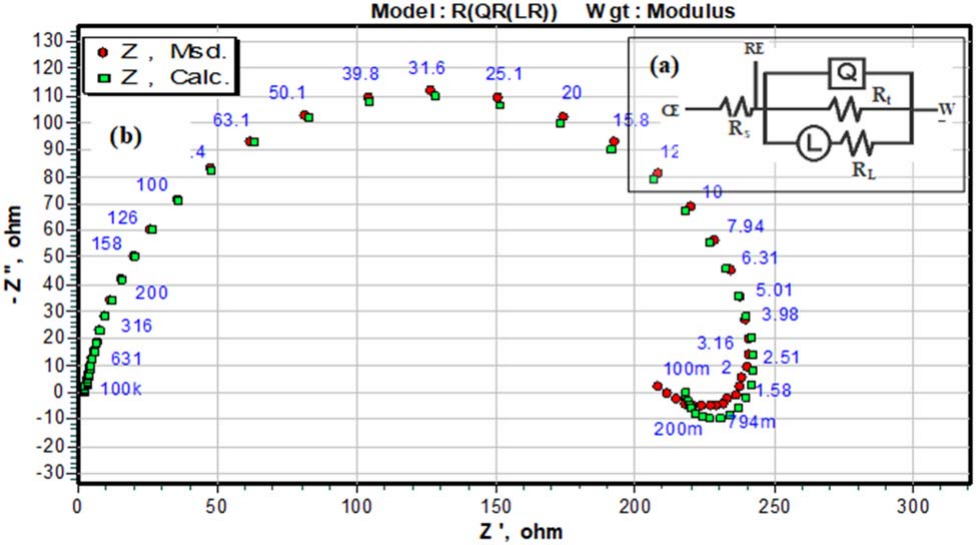
Equivalent circuit model used to fit the experimental impedance data.

Fig. 10 shows the five-element circuit used for impedance simulation. The element *R*_s_ represents the bulk resistance of the medium. The resistance linked to charge transfer is denoted by *R*_ct_. The inductance is modeled *via* the pair *R*_L_ and *L*. A constant phase element, *Q*, is connected across the *R*_t_ and *R*_L_ branches to account for nonideal capacitive behavior. The polarization resistance *R*_p_ can be calculated from eqn (5):5
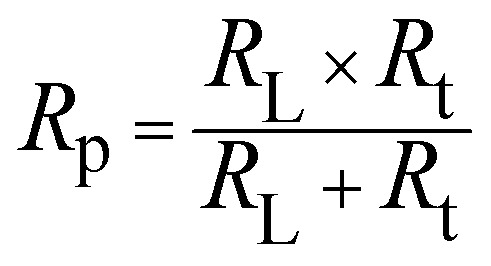


In the equivalent circuit, no ideal capacitor is included. Instead, a constant phase element (CPE) is used to represent the nonideal capacitive behavior of the electrode interface. The CPE accounts for effects such as surface roughness, heterogeneity, and nonuniform current distribution, which prevent the system from behaving like an ideal capacitor.^44,46^ The impedance of the CPE varies with frequency and is expressed as (eqn (6)):6
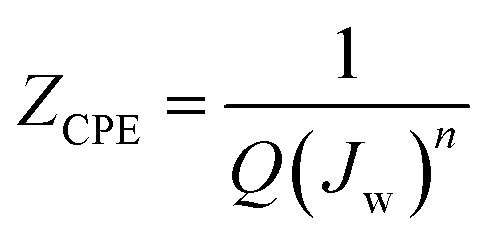
where *Q* is the CPE magnitude (units similar to capacitance), *n* is the phase factor (0 < *n* ≤ 1) indicating deviation from ideal capacitive behavior, *ω* is the angular frequency, and *j* is the imaginary unit.

### 3.6 Interpretation of the Bode plot

**Fig. 11:**
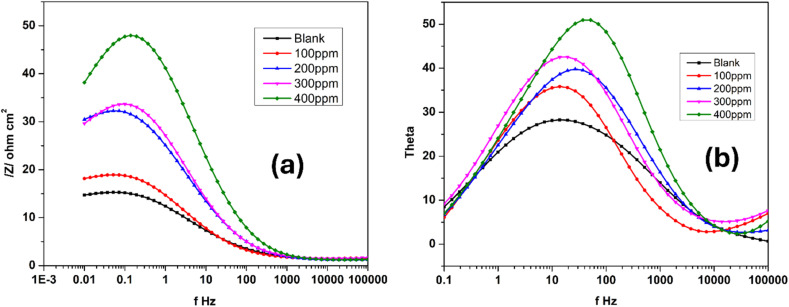
(a) Bode phase and (b) magnitude plots for the alloy in 1.0 M HCl at 30 °C with varying FCND concentrations.

Electrochemical impedance measurements were utilized to obtain Bode plots—both magnitude and phase angle—to evaluate the protective behavior of functionalized carbon nanodots (FCNDs) on maraging steel under acidic conditions. These tests were conducted in a corrosive medium using an open circuit potential (OCP) containing 1 M hydrochloric acid at 30 °C. The results are illustrated in Fig. 11. The phase angle response shows a noticeable upward trend with increasing FCND concentration, reaching a maximum at the most effective inhibitor dosage. This increase in the phase angle suggests improved surface coverage and more capacitive behavior, indicative of the formation of a stable and uniform inhibitor film at the metal–electrolyte interface. In the Bode magnitude plot (Fig. 10b), a single linear region is evident across the frequency range for both the uninhibited and inhibited systems. This behavior is characteristic of systems dominated by one primary electrochemical process. The polarization resistance (*R*_p_), which reflects the resistance to charge movement and is related to the integrity of the inhibitor layer, is inferred from the difference in impedance between the higher and lower ends of the frequency spectrum. A greater separation implies greater resistance to corrosion, as it accounts for the combined effects of surface passivation, metal dissolution, and the barrier characteristics of the adsorbed FCND layer.^47^ The observed increase in the phase angle and *R*_p_ values with increasing FCND loading highlights the material's capacity to hinder electrochemical activity and reinforces its potential as an effective corrosion inhibitor.

#### 3.6.1 Thermodynamic parameters

**Fig. 12:**
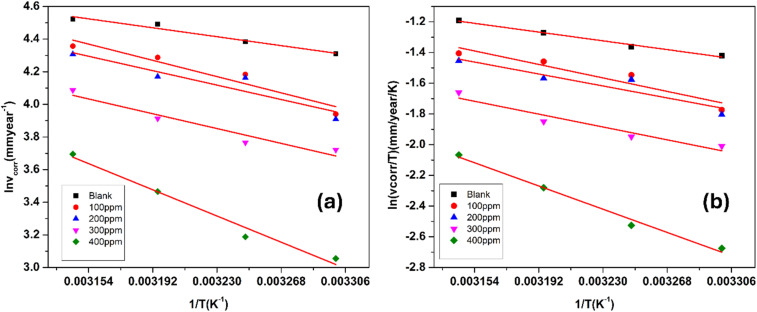
(a) Arrhenius plots showing the effect of temperature on the corrosion rate of maraging steel in 1.0 M HCl containing different concentrations of FCND. (b) Plot of ln(*ϑ*_corr_/*T*) *versus* 1/*T* for the corrosion of maraging steel in 1.0 M hydrochloric acid containing different concentrations of FCND.

The thermodynamic data related to the corrosion inhibition process are summarized in Table 5. These values were calculated on the basis of temperature-dependent corrosion rate (CR) measurements *via* the Arrhenius approach, where ln(CR) was plotted against 1/*T*. This linear relationship, depicted in Fig. 12a, follows the general form of the Arrhenius eqn (7):7
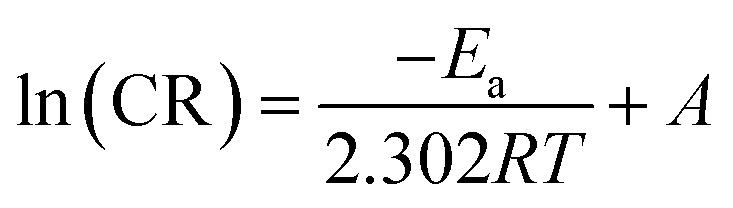


The term *E*_a_ indicates the corrosion activation energy, *R* is the gas constant, and *A* is the preexponential term in the Arrhenius equation. The straight-line trend observed in the Arrhenius plots confirms that the corrosion rate decreases with increasing temperature, which is consistent with thermally activated processes. The calculated slopes of these plots were used to estimate the *E*_a_ values, which are detailed in Table 4. A comparison between the blank and inhibited systems revealed that the activation energy was greater when the inhibitor (FCND) was present. This suggests that the FCND molecules adsorb onto the steel surface, forming a protective barrier that increases the energy requirement for corrosion to occur.^48^ The elevated *E*_a_ values indicate a shift toward reduced metal dissolution in the presence of the inhibitor. Additionally, activation energy values exceeding 20 kJ mol^−1^ typically point toward a physisorption mechanism, where the interaction between the inhibitor molecules and the metal surface is primarily through weak, noncovalent forces. This is consistent with the behavior observed here. The data also indicate that as the concentration of FCND increases, the corresponding activation energy increases, which implies enhanced surface coverage and increased inhibition efficiency. However, at higher temperatures, partial desorption of the physically adsorbed inhibitor may occur, slightly reducing the protection and leading to variations in the *E*_a_ values.^49^

**Table 4:** Activation parameters for the corrosion of maraging steel in 1.0 M HCl with various concentrations of the inhibitor (FCND)

Inhibitor concentration (ppm)	*E*_a_ (kJ mol^−1^)	Δ*H*_a_(kJ mol^−1^)	Δ*S*_a_(kJ mol^−1^)	*E*_a_ − Δ*H*_a_ (kJ mol^−1^)
Blank	14.48	11.98	−168.19	2.5
100	21.78	19.21	−148.48	2.57
200	19.34	17.03	−155.88	2.31
300	20.86	18.31	−153.80	2.55
400	35.15	33.12	−110.65	2.03

#### 3.6.2 Transition state analysis

To further understand the energetics of the corrosion inhibition process, transition state theory was applied. This involved plotting ln (CR/*T*) *versus* 1/*T*, as shown in Fig. 12(b). The relationship follows transition state eqn (8):8
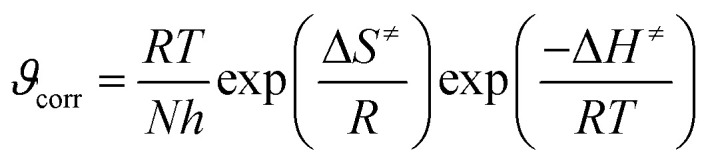
In this context, *R* denotes the universal gas constant, *T* represents the absolute temperature, *N* represents Avogadro's number, *h* indicates Planck's constant, and Δ*H*^≠^ and Δ*S*^≠^ correspond to the activation enthalpy and activation entropy, respectively. The values for Δ*H*^≠^ and Δ*S*^≠^ were obtained from the slope and intercept of the graph plotting ln(CR/*T*) against 1/*T*. The positive Δ*H*^≠^ values suggest that the dissolution of maraging steel is an endothermic process, indicating that the reaction requires energy input, which is consistent with inhibitor-mediated processes. Furthermore, the close match between the activation energy (*E*_a_) and the activation enthalpy (Δ*H*^≠^), differing by approximately 2.5 kJ mol^−1^, supports the theoretical relationship 9:

9 *E*_a_ − Δ*H*_a_ = *RT*

This small difference confirms the validity of transition state theory in this system and suggests a unimolecular reaction pathway for the dissolution process.^50^ This mechanism implies that the reaction proceeds *via* a single activated complex without the need for simultaneous collision between multiple species.

The notably low values of activation entropy (Δ*S*^≠^), observed both in the absence and presence of the inhibitor, suggest that the creation of the activated complex in the rate-limiting step is characterized by an associative mechanism rather than a dissociative mechanism. This results in a decrease in disorder as the system moves from the reactants to the activated complex^51^.

#### 3.6.3 Adsorption isotherm

Adsorption refers to the process where molecules accumulate on a solid surface, separating them from other phases. In corrosion inhibition, organic inhibitor molecules attach themselves to the metal surface by displacing water molecules previously adsorbed there. The degree to which these inhibitors adsorb at the metal–solution interface depends on several factors, including the electrochemical potential, the characteristics of the surface charge, the molecular structure of the inhibitor, and the composition of the surrounding solution. To model the relationship between the fraction of the surface covered by the inhibitor (*θ*) and its concentration (*C*, expressed in mol dm^−1^^3^), various adsorption isotherms, such as the Langmuir, Temkin, Frumkin, and Freundlich isotherms, are commonly applied. These isotherms provide mathematical frameworks that link surface coverage to the inhibitor concentration.^52^

Langmuir adsorption isotherm, linear graph of 
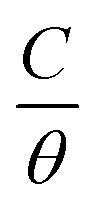
 against *C*10
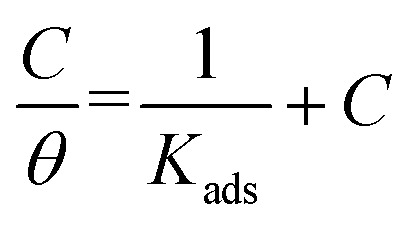


Temkin adsorption isotherm equation, linear graph of *θ vs.* log *C*11
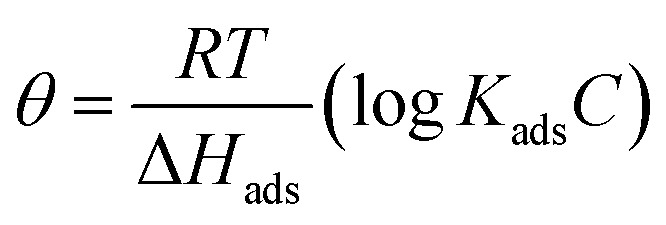


Frumkin adsorption isotherm, linear graph of 
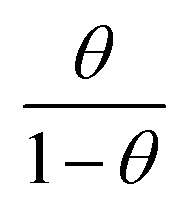
*vs.* log *C*12
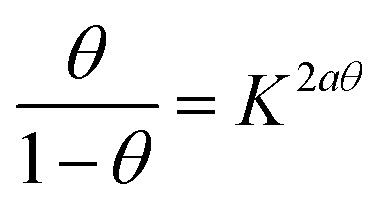


Freundlich isotherm, linear graph of log *θ vs.* log *C*13
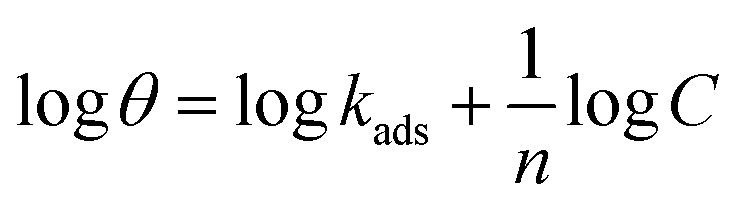


**Fig. 13:**
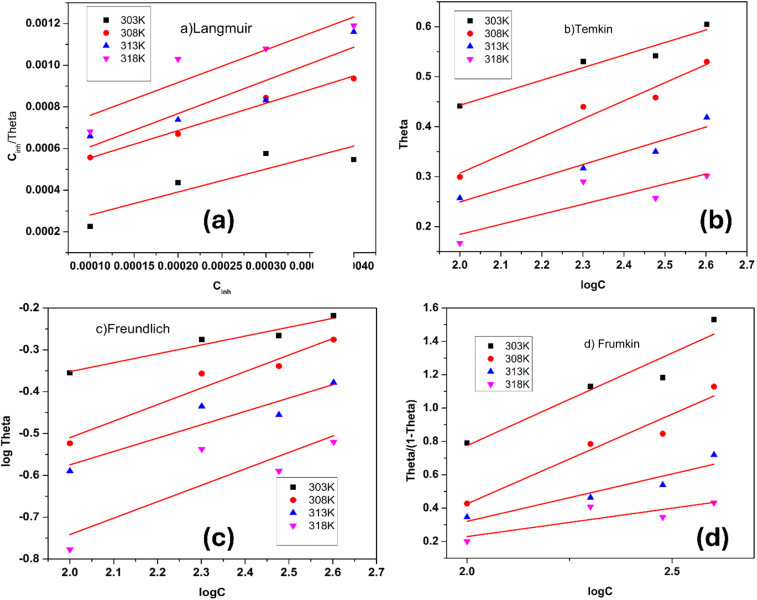
Adsorption isotherms for FCND at various temperatures in 1 M HCl: (a) Langmuir, (b) Temkin, (c) Freundlich, and (d) Frumkin models.

Fig. 13 shows the linear fits corresponding to the adsorption isotherms described by eqn (9)–(12). The coefficient of determination (*R*^2^) values for each isotherm are summarized in Table 5. Among these isotherms, the Langmuir isotherm has the highest *R*^2^ value, indicating that it best represents the adsorption behavior of the system. However, the linear plots do not pass through the origin and have an average correlation coefficient of 0.9235, suggesting a slight deviation from the ideal Langmuir adsorption for the FCND molecules. The positive enthalpy of adsorption suggests that the process of adsorption is endothermic.^50^ On the basis of the Langmuir model, the adsorption sites on the metal surface have consistent energy levels, enabling the creation of a monolayer of inhibitor molecules while disregarding any interactions among the adsorbed species.^53^ The adsorption equilibrium constant (*K*_ads_) was calculated *via* the Langmuir isotherm. The relationship between *K*_ads_ and the standard free energy of adsorption 
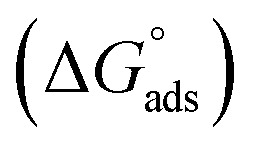
 is represented by eqn (14).

14 Δ*G*  = −2.303*RT* log(55.5*K*_ads_)

where *R* denotes the universal gas constant and where 55.5 mol L^−1^ corresponds to the molar concentration of water. A greater *K*_ads_ value indicates that the adsorption process is proceeding predominantly in the forward direction.

**Table 5:** Thermodynamic characteristics of FCND inhibitor adsorption on maraging steel

Temp. (K)	Langmuir *C*_inh_*vs. C*_inh_/*θ*						Frumkin, *θ*/1 − *θ vs.* log *C*	Temkin, log C *vs. θ*	Freundlich, log *C vs.* log *θ*
	Slope	*K*_ads_ (mol L^−1)^	*R* ^2^	Δ*G*^0^_ads_ (kJ mol^−1^)	Δ*H*^0^_ads_ (kJ mol^−1^)	Δ*S*^0^_ads_ (J K^−1^ mol^−1^)	*R* ^2^	*R* ^2^	*R* ^2^
303	1.103	5.865 × 10^3^	0.7065	−32.34	−9.17	−197.52	0.8739	0.9234	0.9313
308	1.311	2.358 × 10^3^	0.9805	−32.71	−10.89	−197.52	0.9222	0.9404	0.9251
313	1.594	2.227 × 10^3^	0.8138	−31.92	−13.25	−197.52	0.8483	0.9110	0.7989
318	1.574	1.661 × 10^3^	0.7864	−31.75	−13.08	−197.52	0.5918	0.6099	0.6337

In this study, the measured enthalpy of adsorption 
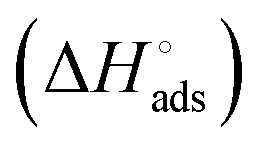
 was found to be less than 20 kJ mol^−1^, indicating that the adsorption of the inhibitor on weld-aged maraging steel occurs primarily through physical adsorption (physisorption) and is an exothermic process. The negative value of the Gibbs free energy of adsorption 
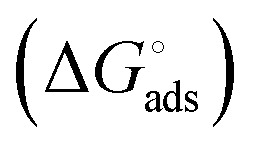
 suggests that the adsorption process proceeds spontaneously with the coating of the inhibitor on the alloy surface, which is found to be inert. Additionally, the notably large negative entropy change 
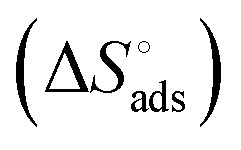
 implies that a reduction in randomness occurs during the shift from reactants to the adsorbed complex on the surface of the alloy, which is consistent with the typical entropy reduction observed in adsorption phenomena.^7^

#### 3.6.4 Surface characterization: atomic force microscopy

**Fig. 14:**
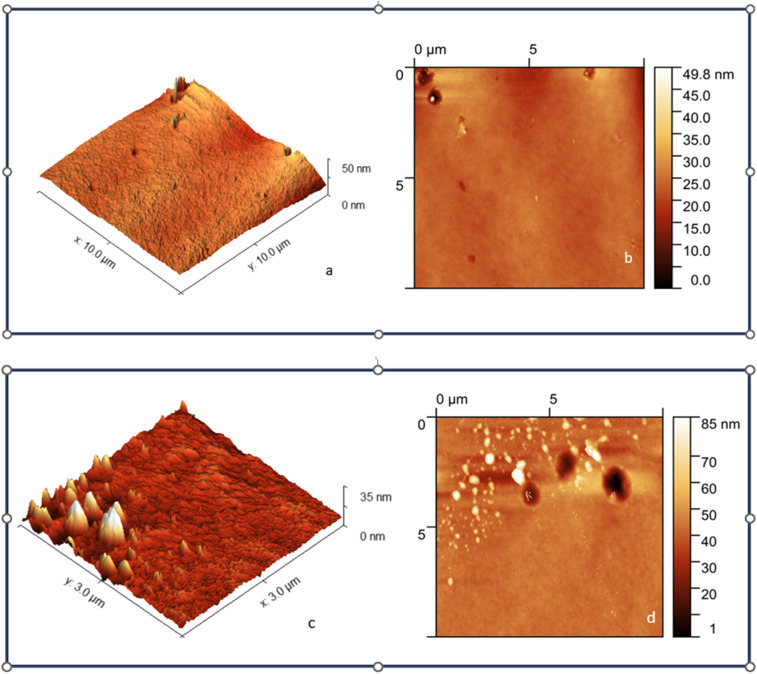
(a–d) AFM micrographs of maraging steel in the presence (a & b) and absence (c & d) of FCNDs at 400 ppm.

The analysis of surface roughness for maraging steel, both with and without the inhibitor, was conducted *via* AFM, providing an effective way to characterize the microstructure. The three-dimensional AFM images are presented in Fig. 14a–d. Fig. 14a and b show that the MS surface appears smoother after exposure to the inhibited solution than after exposure to the uninhibited solution, as shown in Fig. 14c and d, for a duration of 4 hours. The average roughness values for the polished maraging steel in 1.0 M HCl without the inhibitor (Fig. 14c) and with the inhibitor (Fig. 14a) were found to be 438.11 nm and 23.24 nm, respectively. As depicted in Fig. 14c, the electrode surface exhibited a bumpy texture characterized by numerous peaks and valleys due to corrosive acid effects on the surface. The decrease in the RMS roughness from 89.75 nm to 2.49 nm is likely attributed to the formation of an adsorbed layer on the surface.^54^

#### 3.6.5 Corrosion inhibition mechanism

The inhibition process primarily involves the adsorption of functionalized carbon nanodots onto the surface of steel. This adsorption creates a protective layer that serves as a barrier, reducing the direct interaction between the metal and the corrosive hydrochloric acid solution. Consequently, both the anodic dissolution of the metal and the cathodic reduction of hydrogen ions are obstructed, resulting in a significant reduction in the corrosion rate.

In acidic media, positively charged FCNDs also experience electrostatic attraction with negatively charged metal surfaces, which supports physisorption. At elevated temperatures, the adsorption shifts to be dominated by physisorption, as evidenced by the observed adsorption enthalpy 
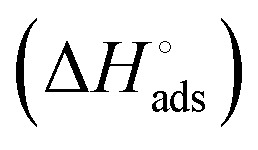
 values below 20 kJ mol^−1^. This synergistic effect suppresses both anodic and cathodic processes, effectively slowing corrosion and extending the service life of steel.

The corrosion process of maraging steel in an acidic environment includes both anodic and cathodic reactions. The schematic representation of the inhibition mechanism is as follows:
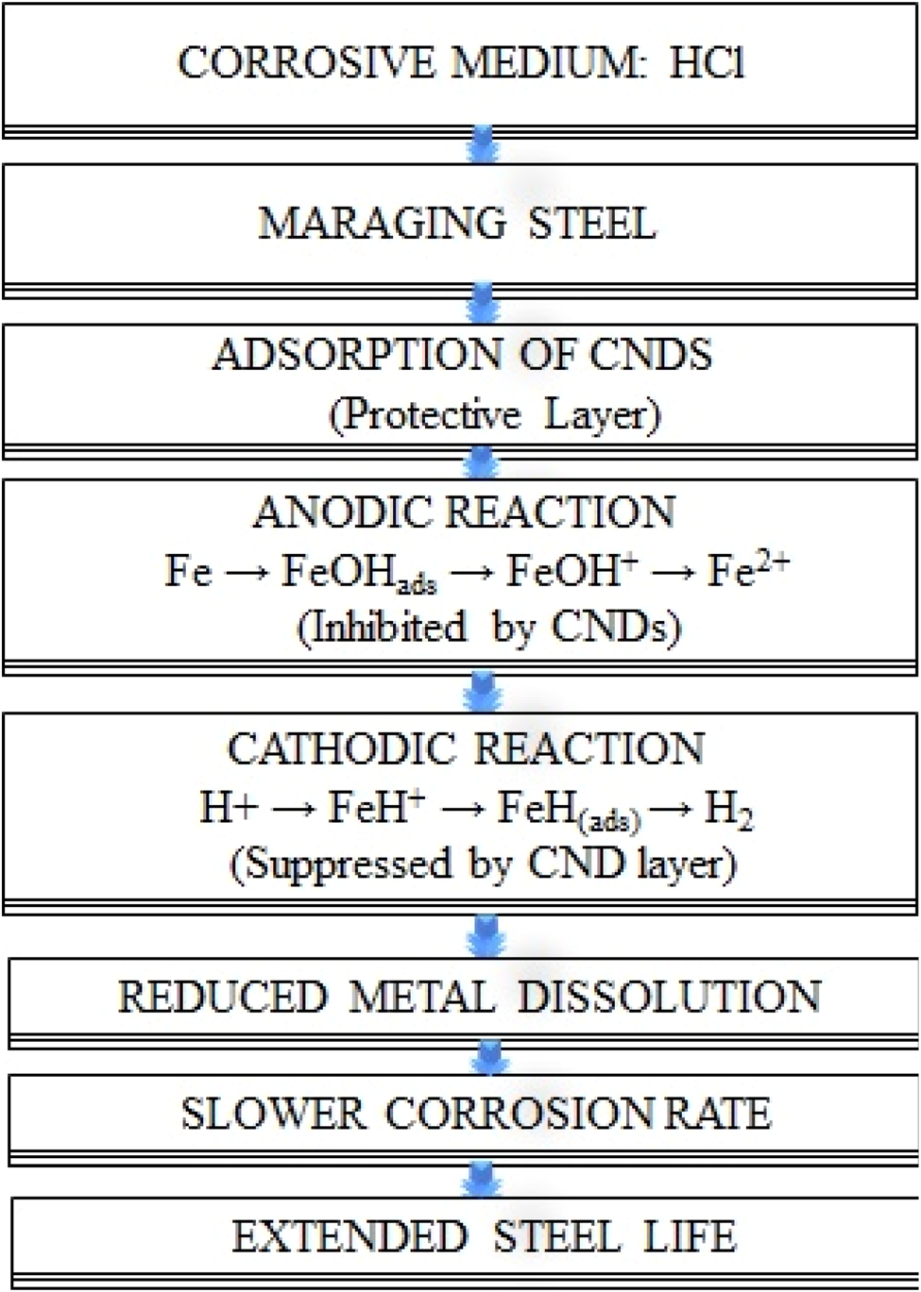


At the anode, iron interacts with water to produce an adsorbed iron hydroxide compound while releasing protons and electrons:

15 (i) Fe + H_2_O ⇌ FeOH_ads_ + H^+^ + e^−^

The adsorbed iron hydroxide then undergoes a rate-determining step, leading to the formation of FeOH^+^ ions:

16 (ii) FeOH_ads_ → FeOH^+^ + e^−^

These ions further react with protons to produce ferrous ions Fe^2+^ and water:

17 (iii) FeOH^+^ + H^+^ → Fe^2+^ + H_2_O

In the reduction process on the cathodic side, hydrogen ions adsorb onto the metal surface, leading to the formation of FeH^+^ species:

18 (iv) Fe + H^+^ → FeH^+^ (ads)

Following this, electron transfer reduces FeH^+^ to produce adsorbed atomic hydrogen:

19 (v) FeH^+^ + e^−^ → FeH (ads)

The adsorbed hydrogen then combines with additional protons and electrons to form molecular hydrogen gas, which is released from the surface:

20 (vi) FeH (ads) + H^+^ +e^−^ → Fe + H_2_

This series of reactions leads to the dissolution of metal at the anode and the release of hydrogen gas at the cathode, which defines the corrosion process in an acidic setting.

## 4. Conclusion

This study verified that carbon nanodots derived from phenylalanine and citric acid serve as effective corrosion inhibitors for maraging steel in a 1.0 M hydrochloric acid environment. Electrochemical impedance and polarization analyses indicate that these nanodots create an adsorbed protective layer on the metal surface, significantly decreasing its interaction with the acidic medium. The inhibition efficiency increased to 73% and improved further with increasing concentrations of the nanodots, demonstrating a clear dependence of corrosion protection on the inhibitor dosage.

## Conflicts of interest

There is no conflict to declare.

## Data Availability

Data will be shared upon request to the authors.
